# PRC1 promotes GLI1-dependent osteopontin expression in association with the Wnt/β-catenin signaling pathway and aggravates liver fibrosis

**DOI:** 10.1186/s13578-019-0363-2

**Published:** 2019-12-16

**Authors:** Shenzong Rao, Jie Xiang, Jingsong Huang, Shangang Zhang, Min Zhang, Haoran Sun, Jian Li

**Affiliations:** 1 Department of Transfusion, Union Hospital, Tongji Medical College, Huazhong University of Science and Technology, Wuhan, 430022 China; 2 Department of Laboratory Medicine, Wuhan Medical Treatment Center, Wuhan City, 430023 Hubei Province China; 3 Department of Transfusion, Xiang’an Hospital of Xiamen University, School of Medicine, Xiamen University, No. 2000 Xiangan Eastroad, Xiangan District, Xiamen, 361101 China; 4 Department of Rehabilitation Medicine, Xiang’an Hospital of Xiamen University, School of Medicine, Xiamen University, No. 2000 Xiangan Eastroad, Xiangan District, Xiamen, 361101 China

**Keywords:** PRC1, GLI, Osteopontin, Wnt/β-catenin, HSC, Liver fibrosis

## Abstract

**Background:**

PRC1 (Protein regulator of cytokinesis 1) regulates microtubules organization and functions as a novel regulator in Wnt/β-catenin signaling pathway. Wnt/β-catenin is involved in development of liver fibrosis (LF). We aim to investigate effect and mechanism of PRC1 on liver fibrosis.

**Methods:**

Carbon tetrachloride (CCl_4_)-induced mice LF model was established and in vitro cell model for LF was induced by mice primary hepatic stellate cell (HSC) under glucose treatment. The expression of PRC1 in mice and cell LF models was examined by qRT-PCR (quantitative real-time polymerase chain reaction), western blot and immunohistochemistry. MTT assay was used to detect cell viability, and western blot to determine the underlying mechanism. The effect of PRC1 on liver pathology was examined via measurement of aspartate aminotransferase (AST), alanine aminotransferase (ALT) and hydroxyproline, as well as histopathological analysis.

**Results:**

PRC1 was up-regulated in CCl_4_-induced mice LF model and activated HSC. Knockdown of PRC1 inhibited cell viability and promoted cell apoptosis of activated HSC. PRC1 expression was regulated by Wnt3a signaling, and PRC1 could regulate downstream β-catenin activation. Moreover, PRC1 could activate glioma-associated oncogene homolog 1 (GLI1)-dependent osteopontin expression to participate in LF. Adenovirus-mediated knockdown of PRC1 in liver attenuated LF and reduced collagen deposition.

**Conclusions:**

PRC1 aggravated LF through regulating Wnt/β-catenin mediated GLI1-dependent osteopontin expression, providing a new potential therapeutic target for LF treatment.

## Introduction

Liver fibrosis (LF) is a common pathological process of many chronic liver diseases developing to cirrhosis and liver cancer with high morbidity and mortality rate [1]. The causes of LF include hepatitis b virus, hepatitis c virus [2], alcohol [3], drugs or poisons [4] and some metabolic factors [5]. After liver injury, the wound-healing response will lead to the accumulation of extracellular matrix (ECM) [6], and the continuous ECM accumulation in liver injury would damage normal liver function and leads to LF-cirrhosis-liver cancer [7]. Meanwhile, activated hepatic stellate cells (HSCs) mainly produce a large amount of collagen and ECM during the process of fibrosis [8]. Therefore, considering that there are no effective therapies for LF, effective treatment approaches for inactivation and anti-proliferation of HSCs to LF, as well as the underlying regulatory mechanisms of LF, are desperately needed.

Microtubule (MT) is crucial for cell growth, cell cycle and migration [9]. The alteration in MT represents a hallmark for chronic liver disease, nonalcoholic steatohepatitis [10], and MT has been recently considered as novel target for cystic fibrosis [11], kidney ischemia/reperfusion injury [12] and renal fibrosis [13]. Protein regulator of cytokinesis 1 (PRC1) has been shown to be a MT-associated regulator of mitosis via binding with MT and facilitate for cytokinesis at telophase [14]. Moreover, PRC1 is also a substrate of CDK (cyclin-dependent kinase) to regulate cell cycle [15]. Therefore, PRC1 is widely known as prognostic biomarker in lung squamous cell carcinoma [16], bladder cancer [14] and breast cancer [17], which could promote tumor cell proliferation and migration. Recently, knockdown of PRC1 was shown to inhibit cell proliferation of hepatocellular carcinoma [18]. Therefore, we speculated that PRC1 might be involved in regulation of LF.

Several signaling pathways have been shown to participate in LF progression [19]. Wnt/β-catenin could transmit inhibition signal to maintain HSC in a quiescent state [20], thus being regarded as the most important pathway. Activation of Wnt/β-catenin could result in HSC activation [21] and promote HSC cell proliferation, finally leading to ECM accumulation and LF [22]. Moreover, suppression of Wnt/β-catenin could attenuate LF [20, 23], suggesting that Wnt/β-catenin might be a novel therapeutic target in LF. Recently, PRC1 was shown to promote malignant properties of hepatocellular carcinoma via activation of Wnt/β-catenin signaling pathway [24]. Whether the regulation ability of PRC1 on LF is dependent on Wnt/β-catenin signaling pathway needs to be investigated.

Therefore, we scoped to investigate the role of PRC1 in HSCs proliferation and Wnt/β-catenin signaling pathway to evaluate the involvement of PRC1 in LF. Our study would enrich the molecule understanding for the pathogenesis of LF and inspire a possible new strategy for preventing LF.

## Results

### PRC1 was up-regulated in CCl_4_-induced mice LF

**Fig. 1:**
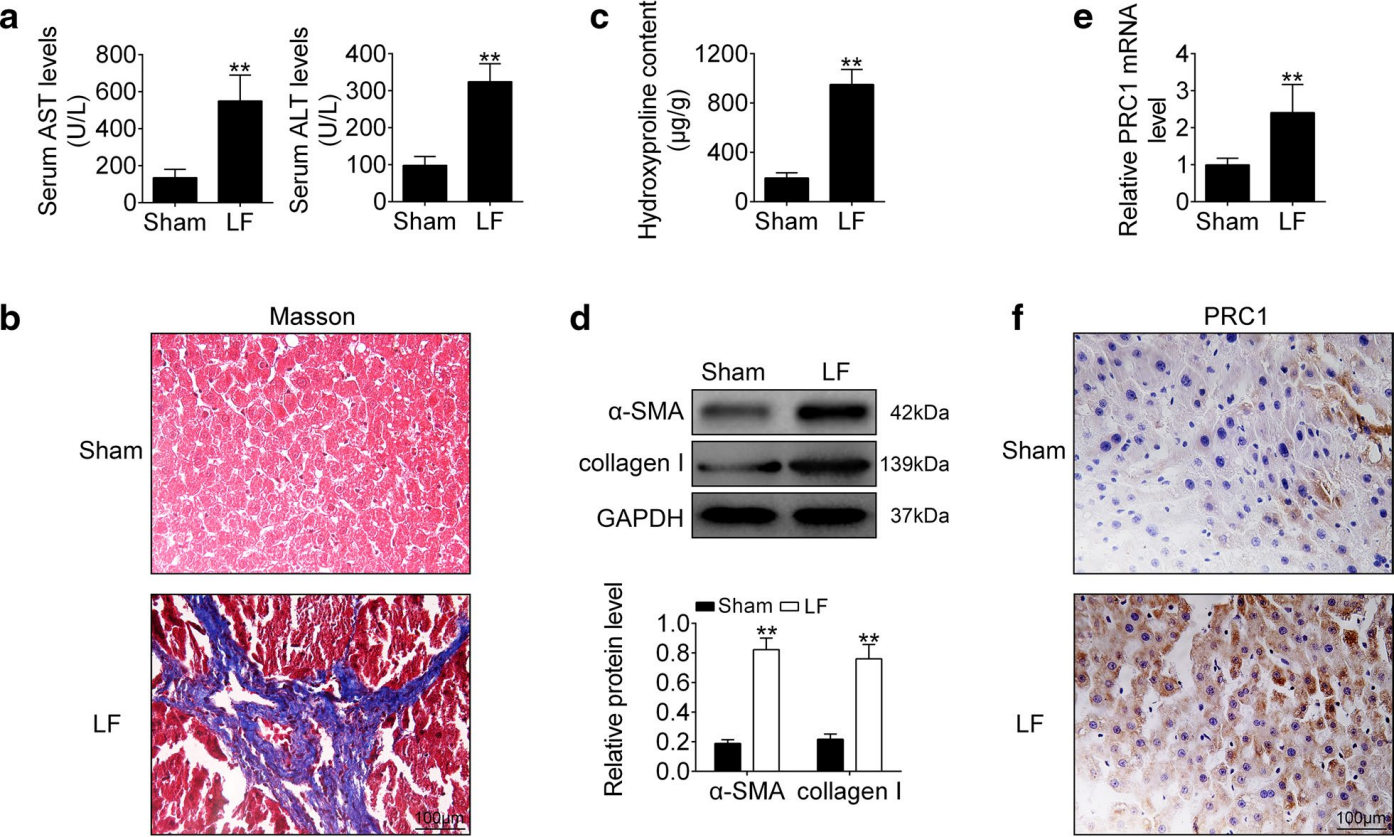
PRC1 was up-regulated in CCl_4_-induced mice LF. **a** Plasma levels of AST and ATL in CCl_4_-induced mice (LF) and the mice without CCl_4_ treatment (Sham). **Represents LF vs. Sham, *p* < 0.01. **b** Masson staining of liver tissues from LF and Sham mice. **c** Quantitative analysis of liver Hyp content from LF and Sham mice. **Represents LF vs. Sham, *p* < 0.01. **d** Proteins expression of α-SMA and type I collagen of liver tissues from LF and Sham mice. **Represents LF vs. Sham, *p* < 0.01. **e** mRNA expression of PRC1 in liver tissues from LF and Sham mice. **Represents LF vs. Sham, *p* < 0.01. **f** Immunohistochemistry analysis of PRC1 in in liver tissues from LF and Sham mice

To determine regulation ability of PRC1 in LF, mice model via CCl_4_ treatment was established. Firstly, plasma levels of AST and ATL were dramatically increased in mice under CCl_4_ treatment (Fig. 1a). Secondly, Masson staining showed that collagen fiber was increased in CCl_4_ treated mice than that in normal mice (Fig. 1b). Moreover, as shown in Fig. 1c, Hyp content analysis indicated an evaluation in collagen content in the livers of mice under CCl_4_ treatment. The increased levels of α-SMA and type I collagen were also confirmed in CCl_4_ treated mice (Fig. 1d). Lastly, qRT-PCR (Fig. 1e) and immunohistochemistry (Fig. 1f) analysis revealed an up-regulation of PRC1 in the livers of mice under CCl_4_ treatment, suggesting that PRC1 may be involved in LF progression.

### PRC1 was up-regulated in activated HSCs

**Fig. 2:**
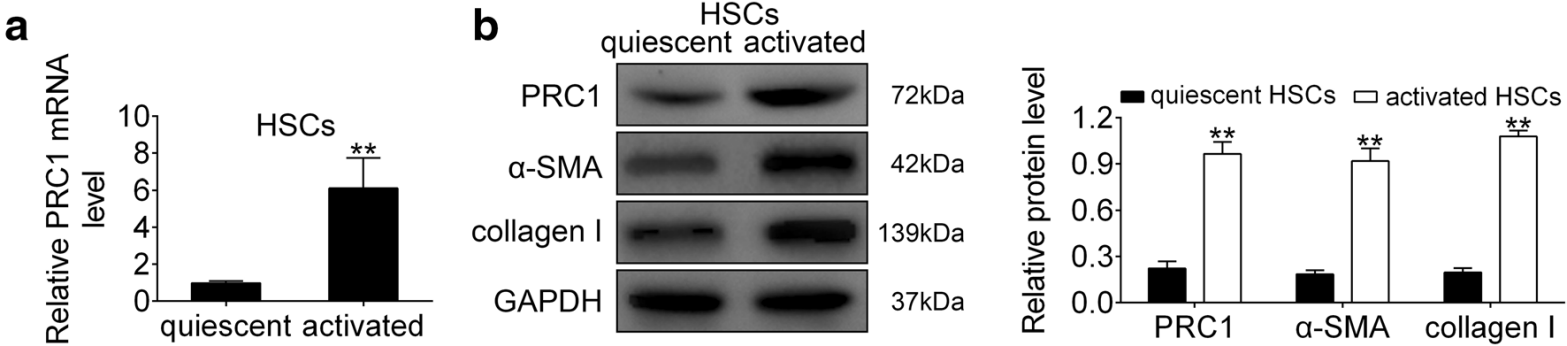
PRC1 was up-regulated in activated HSCs. **a** mRNA expression of PRC1 in quiescent and activated HSCs. **Represents quiescent vs. activated, *p* < 0.01. **b** Proteins expression of PRC1, α-SMA and type I collagen in quiescent and activated HSCs. **Represents quiescent vs. activated, *p* < 0.01

To further investigate expression change of PRC1 during HSC activation, quiescent HSCs were isolated, and then mimicked the in vivo activation process to produce activated HSCs. qRT-PCR (Fig. 2a) and western blot (Fig. 2b) analysis revealed that PRC1 was evidently up-regulated in activated HSCs compared to quiescent HSCs. Moreover, the expression levels of α-SMA and type I were positive correlated with elevation of PRC1 (Fig. 2b), suggesting that PRC1 may be involved in HSCs activation.

### Knockdown of PRC1 suppressed cell proliferation and promoted cell apoptosis of activated HSCs

**Fig. 3:**
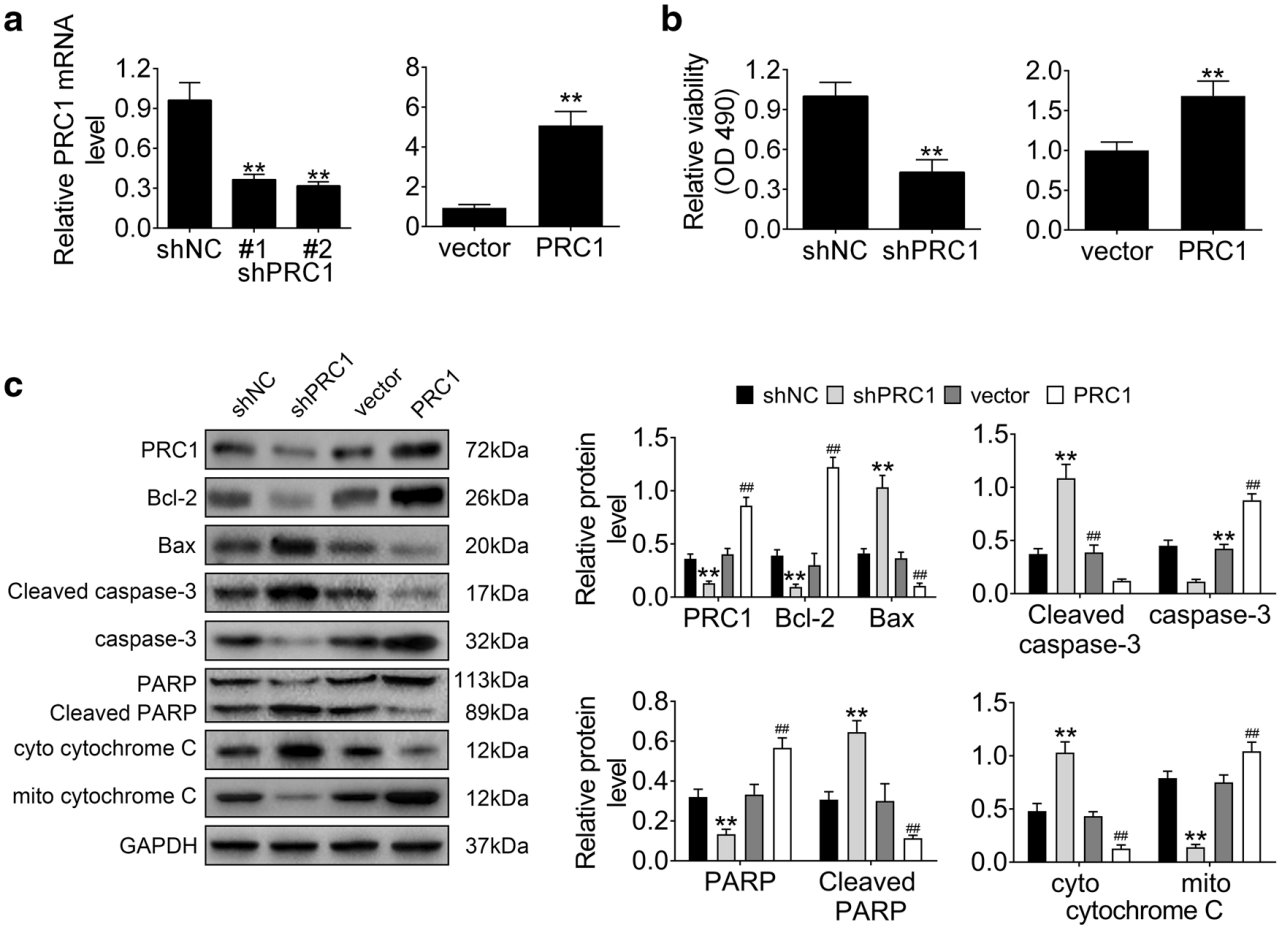
Knockdown of PRC1 suppressed cell proliferation and promoted cell apoptosis of activated HSCs. **a** Transfection efficiency of shPRC1 #1/#2 or pcDNA3.1-PRC1 in activated HSCs. **Represents shPRC1 #1 or #2 vs. shNC; pcDNA3.1-PRC1 (PRC1) vs. pcDNA3.1 (vector) *p* < 0.01. **b** The effects of shPRC1 or pcDNA3.1-PRC1 on cell viability of activated HSCs. **Represents shPRC1 vs. shNC; PRC1 vs. vector, *p* < 0.01. **c** The effects of shPRC1 or pcDNA3.1-PRC1 on proteins expression of Bcl-2, Bax, Cleaved Caspase-3, caspase-3, PARP, Cleaved PARP, cyto cytochrome C and mito cytochrome C in activated HSCs. **Represents shPRC1 vs. shNC, *p* < 0.01. ##Represents PRC1 vs. vector, *p* < 0.01

To evaluate the effect of PRC1 on cell proliferation of activated HSCs, gain- or loss-of functional assays were conducted via transfection with pcDNA3.1-PRC1 or shRNAs targeting PRC1, respectively. Firstly, the transfection efficiency of pcDNA3.1-PRC1 and shPRC1 #1/#2 were confirmed in Fig. 3a. Meanwhile, shPRC #2 with lower expression of PRC1 was selected for the following experiments and named as shPRC1. Secondly, MTT assay showed that knockdown of PRC1 decreased cell viability of activated HSCs (Fig. 3b), while over-expression of PRC1 increased cell viability (Fig. 3b). Lastly, proteins involved in cell apoptosis were detected by western blot analysis. Bcl-2, caspase-3, PARP and mitochondrial cytochrome C (mito cytochrome C) were decreased in activated HSCs transfected with shPRC1, while Bax, Cleaved Caspase-3, Cleaved PARP, cytosol cytochrome C (cyto cytochrome C) were increased (Fig. 3c). Morevoer, over-expression of PRC1 demonstrated the reversed effects on protein expression (Fig. 3c). These results revealed that knockdown of PRC1 suppressed cell proliferation and promoted cell apoptosis of activated HSCs, thus might attenuate LF.

### Knockdown of PRC1 attenuated LF progression

**Fig. 4:**
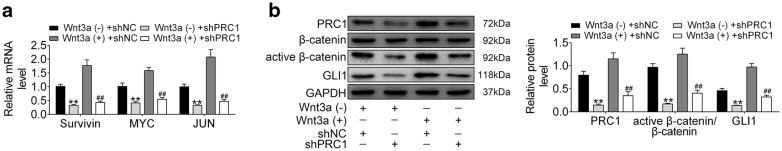
Knockdown of PRC1 attenuated LF progression. **a** The effect of tail vein injection of Ad-shPRC1 on plasma levels of AST and ATL in mice liver tissues. **Represents LF + Ad-shNC vs. Sham, *p* < 0.01. ##LF + Ad-shPRC1 vs. LF + Ad-shNC, *p* < 0.01. **b** Masson staining of liver tissues from Sham mice and LF mice injected with Ad-shNC or Ad-shPRC1. **c** Quantitative analysis of liver Hyp content from Sham mice and LF mice injected with Ad-shNC or Ad-shPRC1. **Represents LF + Ad-shNC vs. Sham, *p* < 0.01. ##LF + Ad-shPRC1 vs. LF + Ad-shNC, *p* < 0.01. **d** mRNA expression of PRC1 in liver tissues from Sham mice and LF mice injected with Ad-shNC or Ad-shPRC1. **Represents LF + Ad-shNC vs. Sham, *p* < 0.01. ##LF + Ad-shPRC1 vs. LF + Ad-shNC, *p* < 0.01. **e** Immunohistochemistry analysis of PRC1 in in liver tissues from Sham mice and LF mice injected with Ad-shNC or Ad-shPRC1. **f** Proteins expression of α-SMA, type I collagen, PRC1, GLI1 and osteopontin of liver tissues from Sham mice and LF mice injected with Ad-shNC or Ad-shPRC1. **Represents LF + Ad-shNC vs. Sham, *p* < 0.01. ##LF + Ad-shPRC1 vs. LF + Ad-shNC, *p* < 0.01

Tail vein injection with adenovirus for knocking down of PRC1 was conducted to explore the clinical application of PRC1 on LF. Firstly, the promoted plasma levels of AST and ATL by LF were decreased by Ad-shPRC1 injection (Fig. 4a). Secondly, Masson staining showed that the increased collagen fiber in LF mice injected with Ad-shNC was decreased in LF mice injected with Ad-shPRC1 (Fig. 4b). Moreover, the increased Hyp content was also decreased Ad-shPRC1 injection (Fig. 4c). Lastly, qRT-PCR (Fig. 4d) and immunohistochemistry (Fig. 4e) analysis revealed that the up-regulation of PRC1 in the livers of LF mice injected with Ad-shNC were down-regulated by Ad-shPRC1 injection, suggesting that knockdown of PRC1 attenuated LF progression. Furthermore, the increased levels of α-SMA, type I collagen, PRC1, GLI1 and osteopontin in LF mice injected with Ad-shNC were decreased in LF mice injected with Ad-shPRC1 (Fig. 4f).

### PRC1 regulated GLI1 expression in association with the Wnt/β-catenin signaling pathway

**Fig. 5:**
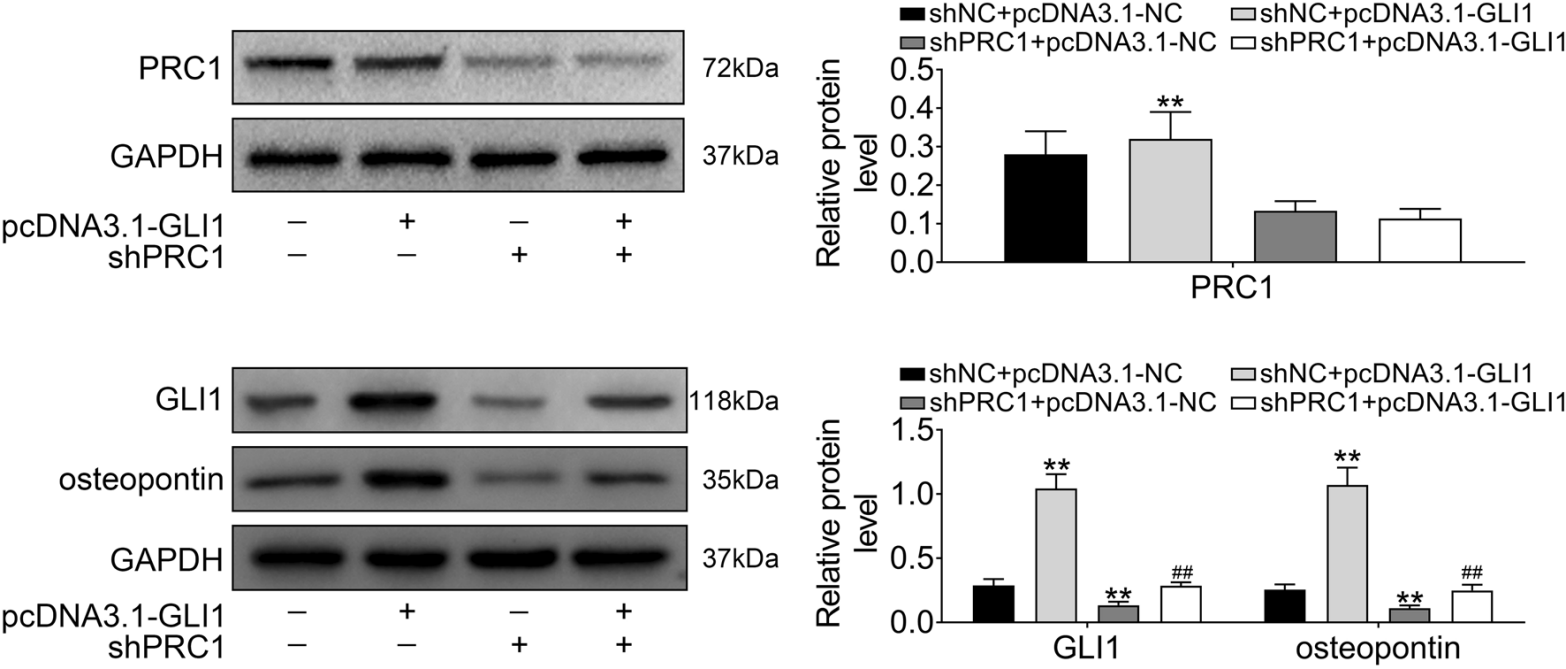
PRC1 regulated GLI1 expression in association with the Wnt/β-catenin signaling pathway. **a** The effect of PRC1 and Wnt3a on mRNAs expression of survivin, MYC and JUN in activated HSCs. **Represents Wnt3a (−) + shPRC1 vs. Wnt3a (−) + shNC, *p* < 0.01. ##Represents Wnt3a (+) + shPRC1 vs. Wnt3a (+) + shNC, *p* < 0.01. **b** The effect of PRC1 and Wnt3a on proteins expression of PRC1, β-catenin, active β-catenin and GLI1 in activated HSCs. **Represents Wnt3a (−) + shPRC1 vs. Wnt3a (−) + shNC, *p* < 0.01. ##Represents Wnt3a (+) + shPRC1 vs. Wnt3a (+) + shNC, *p* < 0.01

Wnt/β-catenin is involved in LF progression, the effect of PRC1 on Wnt/β-catenin signaling pathway was then determined to uncover the underlying mechanism involved in the regulation of PRC1 on LF. Low dose of Wnt3a (1.5625 ng/mL) treatment promoted expression of Wnt targets, such as survivin, MYC and JUN in activated HSCs (Fig. 5a), while HSCs transfected with shPRC1 decreased survivin, MYC and JUN (Fig. 5a). Moreover, the expression of survivin, MYC and JUN in activated HSCs transfected with shPRC1 and treated with Wnt3a were restored to that in cells transfected with shPRC1 (Fig. 5a), suggesting a possible correlation between PRC1 and Wnt/β-catenin signaling pathway. Knockdown of PRC1 in activated HSCs decreased active β-catenin (92 kDa)/β-catenin ratio and PRC1 (Fig. 5b). Wnt3a treatment not only induced Wnt/β-catenin signaling pathway activation, as shown by increase of active β-catenin/β-catenin ratio (Fig. 5b), but also promoted PRC1 expression (Fig. 5b). However, additional transfection with shPRC1 in cells under Wnt3a treatment decreased active β-catenin/β-catenin ratio and PRC1 (Fig. 5b), suggesting that PRC1 was dynamically up-regulated by Wnt3a signaling in activated HSCs, and knockdown of PRC1 decreased Wnt target expression and reduced active β-catenin/β-catenin ratio. Since Wnt/β-catenin signaling has been shown to be correlated with hedgehog (Hh) signaling pathway, and activation of Hh signaling contributes to LF, the effect of PRC1 on Hh signaling was then determined. As shown in Fig. 5b, knockdown of PRC1 decreased Hh signaling related protein (GLI1), while Wnt3a treatment increased GLI1 expression. Moreover, additional transfection with shPRC1 in cells under Wnt3a treatment decreased GLI1 expression (Fig. 5b), suggesting that PRC1 regulated GLI1 expression in association with the Wnt/β-catenin signaling pathway in activated HSCs.

### PRC1 promoted GLI1-dependent osteopontin expression

**Fig. 6:**
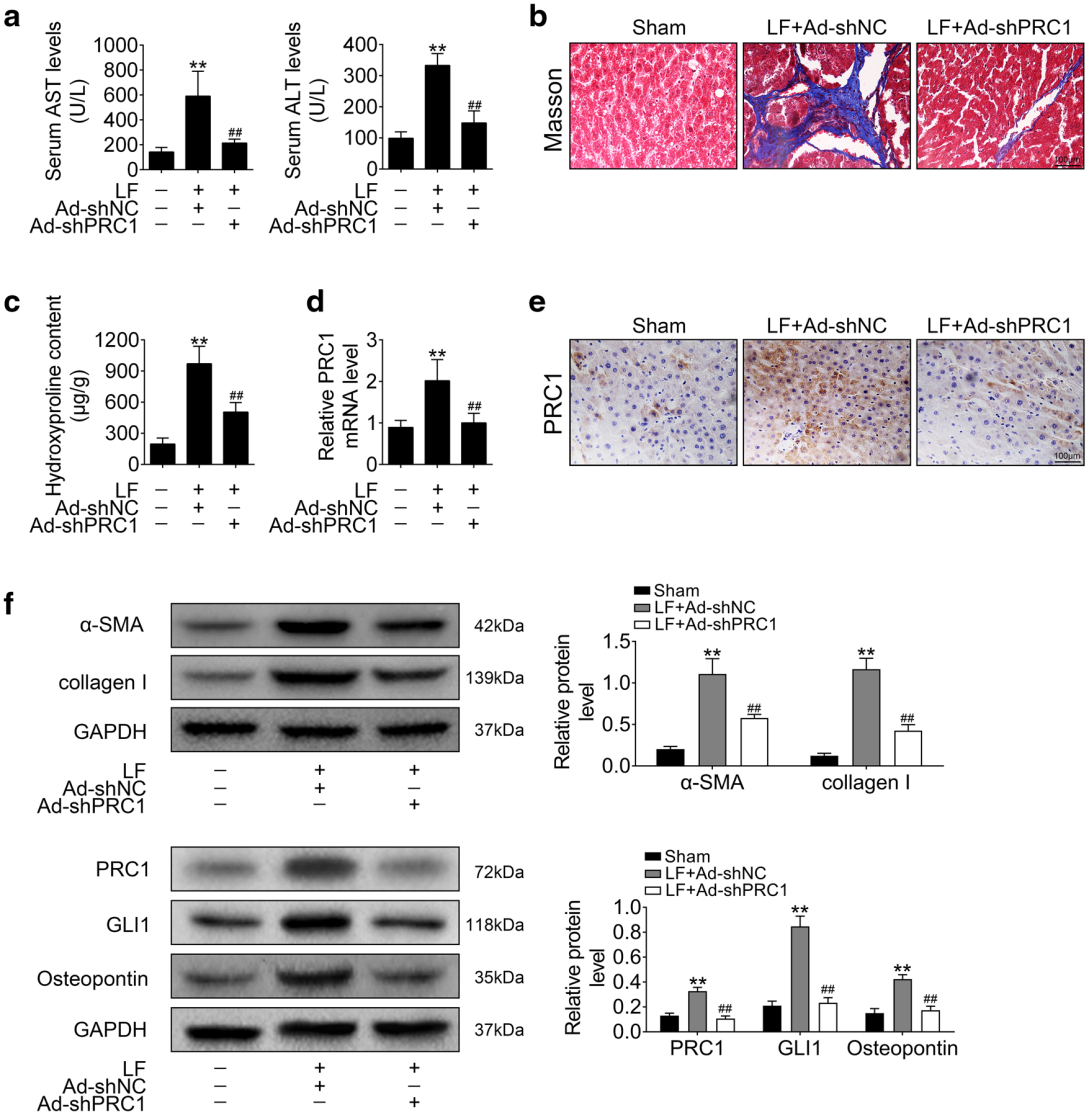
PRC1 promoted GLI1-dependent osteopontin expression. The effect of PRC1 and GLI1 on proteins expression of PRC1, GLI1 and osteopontin in activated HSCs. **Represents pcDNA3.1-GLI1 + shNC vs. pcDNA3.1-NC + shNC, *p* < 0.01. ##pcDNA3.1-GLI1 + shPRC1 vs. pcDNA3.1-NC + shPRC1, *p* < 0.01

As the downstream target of GLI1, osteopontin expression altered by PRC1 in activated HSCs was investigated. Firstly, over-expression of GLI1 had no significant effect on protein expression of PRC1, and shPRC1 significantly decreased PRC1 (Fig. 6). Moreover, over-expression of GLI1 could promote GLI1 and osteopontin expression (Fig. 6), while knockdown of PRC1 decreased GLI1 and osteopontin (Fig. 6). Moreover, activated HSCs transfected with pcDNA3.1-GLI1 and shPRC1 could reversed the promotion ability of GLI1 overexpression on GLI1 and osteopontin expression (Fig. 6), suggesting that PRC1 promoted GLI1-dependent osteopontin expression in activated HSCs.

## Discussion

It has been demonstrated that the progression of LF is greatly influenced by continuous ECM accumulation, which is produced by activated HSCs [25]. PRC1 has been shown to be a critical regulator of proliferation and apoptosis [26], thus may performing decisive roles in regulation of activated HSCs proliferation and LF. We aimed to validate the mechanism underlying the effects of PRC1 on LF.

In this study, we firstly established mice LF model via CCl_4_ treatment, and found out an increase of AST, ATL, Hyp, α-SMA and type I collagen. AST is produced in liver and regulates amino acid metabolism, ALT is mainly found in liver cells. Elevation of AST [27] and ATL [28] have been considered as markers of LF. Moreover, during LF, excessive accumulation of ECM, such as collagen, is the mainly cause [29]. Hyp is the composition of collagen, and also regarded as biomarker of LF [30], as well as α-SMA [31] and type I collagen [32]. Therefore, positive correlated with increase of AST, ATL, Hyp, α-SMA and type I collagen, PRC1 was also up-regulated in CCl_4_-induced mice LF. Knockdown of PRC1 indicated anti-fibrotic effect against LF, as shown by decrease of AST, ATL, Hyp, α-SMA and type I collagen in LF mice injected with Ad-shPRC1.

Other than collagen accumulation, as the major fibrogenic population place, activated HSCs progression is tightly associated with LF progression [33]. The promotion of activated HSCs proliferation contributes to LF progression [34], and the apoptosis of activated HSCs results in alleviation of LF [35]. Here, consistent the in vivo effect of PRC1 against LF, PRC1 was also up-regulated in activated HSCs, and knockdown of PRC1 inhibited cell proliferation and induced cell apoptosis of activated HSCs, thus attenuating LF progression. The intrinsic apoptotic pathway, regulated by Bcl-2 family, participates in apoptosis of activated HSCs during LF [36]. Down-regulation of anti-apoptotic protein, Bcl-2, and up-regulation of pro-apoptotic protein, Bax, contributes to cell apoptosis [36]. Our result showed that knockdown of PRC1 decreased Bcl-2, increased Bax and Cleaved Caspase-3 to promote activated HSCs apoptosis. Hence, knockdown of PRC1 exerted anti-proliferative and pro-apoptotic effects on activated HSCs to attenuate LF.

The underlying mechanism involved in regulation of activated LF progression and LF via PRC1 was then determined. Wnt/β-catenin signaling pathway not only promote the activation and proliferation of HSCs, but also contributes to the development of LF via regulation of fibrosis-related gene expression [37]. Blockage of Wnt/β-catenin contributes to the inhibition of HSCs activation and proliferation, thus alleviating LF [20]. Moreover, PRC1 was recently shown to promote early recurrence of hepatocellular carcinoma in association with Wnt/β-catenin signaling pathway [24]. The present study for the first time revealed that PRC1 was dynamically up-regulated by Wnt3a signaling in activated HSCs, and knockdown of PRC1 decreased Wnt target expression to attenuate LF progression. Moreover, Hh signaling also participates in HSCs activation and proliferation during LF [38]. Inhibitors of Hh pathway functions as candidates for anti-fibrotic therapeutic agents of LF [39]. Here, we indicated that knockdown of PRC1 decreased expression of Hh signaling related protein (GLI1) to inhibit Hh signaling. Furthermore, crosstalk between Wnt and Hh signaling pathways has been widely investigated in cancer [40]. For example, GLI could bind to promoter of Wnt genes to regulate Wnt/β-catenin signaling [41], and β-catenin could enhance GLI1 transcriptional activity [42]. Recently, crosstalk between Wnt and Hh was shown to be involved in pulmonary fibrosis, and GLI1 was proved to be a potential therapeutic target in pulmonary fibrosis [43]. The present study showed that GLI1 expression was promoted by Wnt activation in LF, and additional knockdown of PRC1 could inhibit GLI1 expression. GLI1 functions as transcriptional factor and binds to promoter of osteopontin [44] and the pro-fibrogenic of osteopontin on LF dependents on promotion of HSC activation and ECM deposition [45]. Promotion of osteopontin via Hh activation contributes to fibrosis progression [46]. Our result also showed that knockdown of PRC1 decreased ostepontin expression, suggesting that PRC1 could aggravate LF through regulating Wnt/β-catenin mediated GLI1-dependent osteopontin expression. Moreover, PI3K/AKT signaling pathway is involved in HSC proliferation and apoptosis, and inhibition of PI3K/AKT signaling pathway has been considered as potential therapeutic mechanism for LF treatment [47]. The effect of PRC1 on PI3K/AKT signaling pathway or other pathways involved in HSCs proliferation and apoptosis needs to be further investigated.

## Conclusion

Knockdown of PRC1 not only exerts anti-proliferative and pro-apoptotic effects on activated HSCs, but also exerts anti-fibrosis effect on LF in association with Wnt/β-catenin mediated GLI1-dependent osteopontin expression, suggesting a novel insight into the treatment of LF.

## Materials and methods

### Animal model

All the animal experiments were approved by Ethics Committee of Xiang’an Hospital of Xiamen University, School of Medicine, Xiamen University, and in accordance with Center for Animal Resources and Development regulations for animal care. Sixty male C57BL/6J mice with 7-week-old were obtained from Experimental animal center of huazhong university of science and technology. Mice were randomly separated into two groups: sham group (N = 15) and LF group (N = 45). For LF group, the mice were intraperitoneal injected with 5 μL per g body weight 10% CCl_4_ (Sigma-Aldrich, St. Louis, MO, USA) twice weekly in olive oil for 6 weeks. Sham mice were intraperitoneal injected with the same volume of olive oil as LF group. Two days after the last CCl_4_ injection, mice were anesthetized with 65 mg per kg body weight of sodium pentobarbital. Serum samples were collected and liver tissues were harvested for further analysis.

### Adenovirus injection

Ad-shPRC1, as well as the negative control (Ad-shNC), were constructed by GenePharma (Shanghai, China). Mice of LF group were randomly seperated into three groups: LF group (N = 15), LF with Ad-shNC (N = 15), LF with Ad-shPRC1 (N = 15). For LF with Ad-shNC or Ad-shPRC1 groups, 1 day before CCl_4_ injection, 100 μL 1 × 10^9^ transducing units Ad-shNC or Ad-shPRC1 were injected via the tail vein once. Following 6 weeks treatment with CCl_4_, mice were also anesthetized, and the serum and liver tissues were also collected.

### Biochemical analysis

Serum levels of aspartate aminotransferase (AST) and alanine aminotransferase (ALT) were evaluated via the Automated Biochemical Analyzer (AU-680, Beckman, Germany). For hydroxyproline (Hyp) content analysis, liver tissues were firstly homogenized in Tris–HCl buffer via polytron homogenizer (Kinematical, Lucerne, Switzerland) and then hydrolyzed at 120 °C overnight. The content of Hyp in liver tissues was measured by A030-2 kit (NanJing JianCheng Bioengineering Institute, Nanjing, China).

### Histopathological analysis

Liver tissues were firstly fixed in 10% formalin, and then processed for paraffin embedding and slice into 5 μm sections. The sections were stained with Masson according to standard protocols, and examined under microscope (Olympus Corporation, Tokyo, Japan). For immunohistochemistry analysis, the liver sections were firstly deparaffinized and then rehydrated in a descending alcohol series. After antigen retrieval, the sections were added with 3% H_2_O_2_ and blocked in 10% normal goat serum. Sections were then incubated with primary antibody against PRC1 (1:1000; ab132234, Abcam, Cambridge, UK) overnight. Lastly, sections were incubated with biotinylated goat anti-rabbit IgG antibody, followed by incubation with peroxidase-conjugated biotin-streptavidin complex, and stained with diaminobenzidine. The sections were counterstained with hematoxylin, and photographed under microscope.

### Cell culture

Primary mice HSCs were isolated as before [48] via in situ pronase/collagenase perfusion of mouse liver followed by in vitro density gradient-based separation. Quiescent HSCs were cultured in Dulbecco’s modified Eagle medium (Gibco, Waltham, MA, USA) complemented with 10% fetal bovine serum and antibiotics under a humidified atmosphere of 5% CO_2_ at 37 °C. For activation of HSCs, cells were culture in Dulbecco’s modified Eagle medium with 1000 mg/L glucose for 3 days.

### Cell transfection

pcDNA3.1-GLI1 and pcDNA3.1-PRC1 were obtained from AxyBio co., LTD (Changsha, China) for the over-expression of GLI1 and PRC1. shRNAs targeting PRC1 (shPRC1 #1 or #2) were synthesized by GenePharma. To investigate the effect of PRC1 on Wnt/β-catenin signaling pathway, HSCs were treated with or without 1.5625 ng/mL wnt3a. HSCs with 1 × 10^6^ cells/well were seeded into 12-well plates and then were transfected with pcDNA3.1-GLI1, pcDNA3.1-PRC1, pcDNA3.1-NC, shPRC1 #1 or #2, negative control (shNC) using Lipofectamine^®^ 3000 (Thermo Fisher, Waltham, MA, USA). Two days transfection, the cells were collected for the following experiments.

### Cell viability

HSCs with 1 × 10^3^ cells/well were seeded in a 96-well plate. MTT cell proliferation assay kit (Beyotime Biotechnology, Jiangsu, China) was used to detect cell viability under microplate reader (Bio-Rad 550, USA) to determine optical density at 490 nm.

### qRT-PCR

Total RNAs were isolated via Trizol reagent, and cDNA was synthesized by Prime Script RT reagent kit. LightCycler DNA Master SYBR Green I Kit (Roche Diagnostics) was used to detect the mRNA expression on the LightCycler system (Roche Diagnostics). 2^−ΔΔCT^ method was used to analyze the relative expression to GAPDH. The primer sequences were as listed in Table 1.

**Table 1:** Primmer sequences

Gene	Sequence
PRC1	Forward: 5′-CCTATTCTGAGTTTGCGAAGGA-3′
	Reverse: 5′-TGATCAGGGCTTCTCAGGAC-3′
survivin	Forward: 5′-CATCTCTACATTCAAGAACTGG-3′
	Reverse: 5′-CCTTGAAGCAGAAGAAACAC-3′
MYC	Forward: 5′-TGAGGAGGAACAAGAAGATG -3′
	Reverse: 5′-ATCCAGACTCTGACCTTTT -3′
JUN	Forward: 5′-CTGTCCCTCTCCACTGCAAC-3′
	Reverse: 5′-AAACACATTAGGCGCAATCC-3′
GAPDH	Forward: 5′-TGCACCACCAACTGCTTAGC-3′
	Reverse: 5′-GGCATGGACTGTGGTCATGAG-3′

### Western blot assay

The isolated proteins (20 μg per lane) from liver tissues or HSCs were separated using 10% sodium dodecyl sulfate–polyacrylamide gelelectrophoresis and transferred onto nitrocellulose membrane (Millipore, Bedford, MA). The membrane was incubated with skimmed milk (5%), and then with the primary antibodies, including anti-PRC1 (Abcam, 1:1000); anti-α-SMA and anti-collagen I (1:1500); anti-Bcl-2, anti-Bax, anti-Cleaved Caspase-3 and anti-caspase-3 (1:2000), anti-β-catenin, anti-activated β-catenin, anti-PARP and anti-Cleaved PARP (1:2500), anti-GLI, anti-osteopontin, anti-cyto cytochrome C, anti-myto cytochrome C and anti-GAPDH (1:3000) at 4 °C overnight. The secondary antibodies (HRP goat anti-rabbit, 1:2000) were applied to incubate the membranes at 37 °C for 120 min. The protein was exposed using ECL detection reagent.

### Statistics analysis

The data were shown as mean ± standard deviation, and statistical analyses were performed via GraphPad Prism 6.0 (GraphPad Software, Inc., La Jolla, CA, USA). Student’s t text was used to compare the difference between two groups, one-way ANOVA with Turkey’s test to compare the difference among multiple groups. *P* < 0.05 was regarded as statistically significant.

## Data Availability

All data generated or analyzed during this study are included in this published article.

## Abbreviations

PRC1: protein regulator of cytokinesis 1
LF: liver fibrosis
CCl_4_: carbon tetrachloride
HSC: hepatic stellate cell
AST: aspartate aminotransferase
ALT: aminotransferase
GLI1: oncogene homolog 1
MT: microtubule
CDK: cyclin-dependent kinase
